# Forgotten Kirschner Wire Causing Severe Hematuria

**DOI:** 10.1155/2014/305868

**Published:** 2014-07-17

**Authors:** Santosh Kumar, Shrawan Kumar Singh, Kumar Jayant, Swati Agrawal, Kalpesh Mahesh Parmar, Sriharsha Ajjoor Shankargowda

**Affiliations:** Department of Urology, Postgraduate Institute of Medical Education and Research, Chandigarh 160012, India

## Abstract

Kirschner wire (K-wire) is commonly used in the treatment of hip fracture and its migration into pelvis leading to bladder injury is a very rare complication. Nonremoval of these devices either because of lack of followup or because of prolonged requirement due to disease process is associated with this complication. We report a case of a patient who presented with acute onset severe hematuria with clot retention secondary to perforation of bladder by a migrated K-wire placed earlier, for the treatment of hip fracture. Initial imaging showed its presence in the soft tissues of the pelvis away from the major vascular structures. Patient was taken for emergency laparotomy and wire was removed after cystotomy. Postoperative period was uneventful and patient was discharged in satisfactory condition. K-wires are commonly used in the management of fracture bones and their migration has been reported in the literature although such migration in the intrapelvic region involving bladder is very rare. Early diagnosis and prompt removal of such foreign bodies are required to avert potentially fatal involvement of major structures.

## 1. Introduction

Pins and wires are commonly used as internal fixator in orthopaedic surgery for management of fractures at various regions in our body. Though, these internal fixation tools (Steinman, Kirschner wires) have a proclivity to migrate. Still migration of K-wire to the intrapelvic region leading to perforating the bladder is an exceptionally rare event [1]. The exact incidence of postoperative pin migration is not known. One of the largest studies was done by Lyons and Rockwood who found 47 cases of wire migration from 1943 to 1981, of which 17 migrated to major vascular structures [2]. This migration when involving a major vascular or a vital intra-abdominal structure as bowel or bladder can result in devastating complications and may be fatal. Thus, early recognition and immediate removal of these implants are of the prime importance in order to ward off such complications. Here, we present a rare case of acute onset hematuria in a 39-year-old female treated for right hip fracture with K-wire which subsequently migrated to intrapelvic region leading to perforation of the bladder.

## 2. Case Report

**Figure 1:**
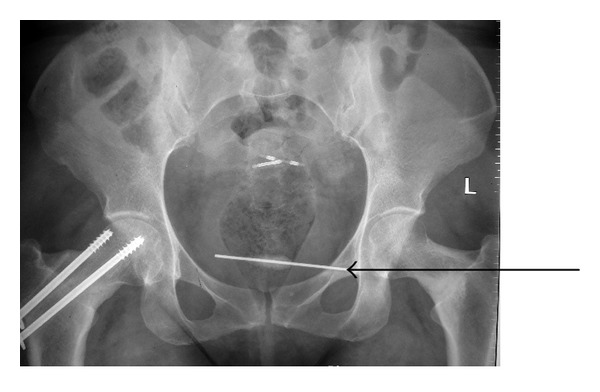
Photograph of the X-ray of pelvis showing the K-wire in the pelvis. A previously inserted copper-T in the uterus is also seen.

**Figure 2:**
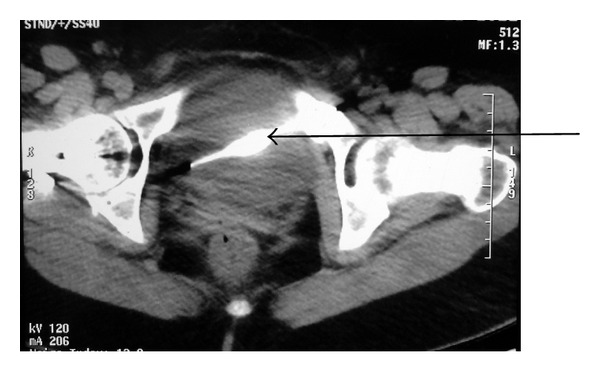
Computed tomography showing K-wire lying in the soft tissues of the pelvis.

**Figure 3:**
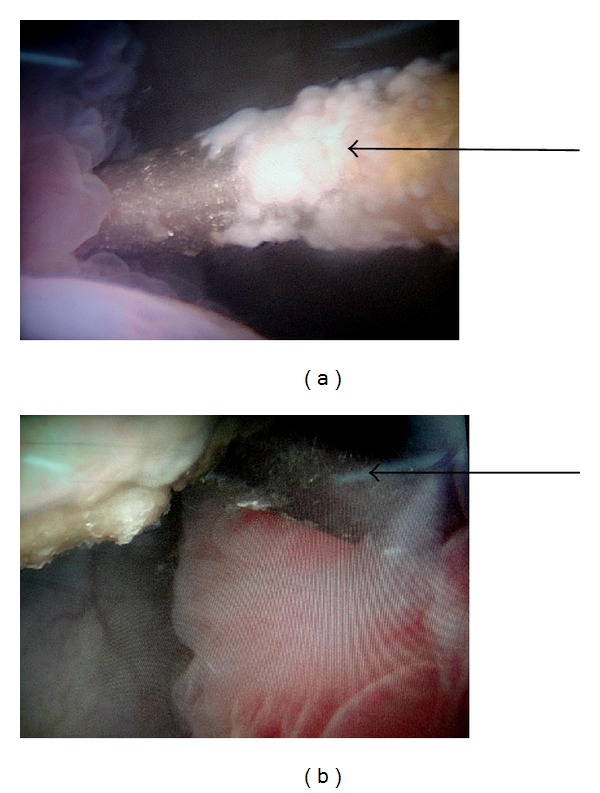
(a) Cystoscopic photograph showing the encrusted K-wire perforating through the right lateral wall of the bladder. (b) Cystoscopic photograph showing the K-wire exiting through the left lateral wall of the bladder.

**Figure 4:**
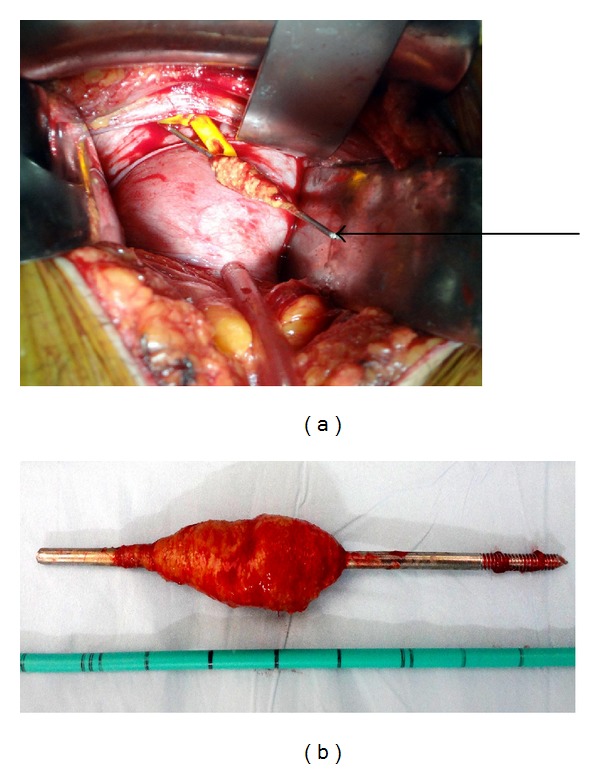
(a) Intraoperative photograph of the K-wire in the bladder. (b) The K-wire after removal from the bladder.

A 39-year-old female presented to the emergency department of our institute with complain of acute onset severe hematuria and clot retention. She had a history of lower abdominal pain on and off with dysuria for the last six months. Her medical records revealed that she had undergone open reduction and internal fixation with a K-wire for right hip fracture two years back. The migration of the wire was reported but removal was not performed because patient was lost to followup. At triage the vital signs were as follows: blood pressure: 90/50 mmHg; heart rate: 116 beats/min; respiratory rate: 22 breaths/min; and temperature (oral): 98.2°F. The patient was ill appearing, grimacing in pain, and clutching her lower abdomen. The abdomen was diffusely tender to palpation and more focally in the suprapubic area. Blood was present at the urethral meatus. She was promptly resuscitated with intravenous fluids and blood was sent for laboratory analysis and crossmatching. She was transfused 3 units of blood. Her initial investigation reports revealed Hb: 6 g/dL, TL: 15,000/*μ*L with predominance of neutrophils, platelet count: 150,000/*μ*L, prothrombin time: 16 seconds, and INR: 1.3. Her serum urea was 46 mg/dL, serum creatinine 0.9 mg/dL, and serum blood sugar 104 mg/dL and ABG showed metabolic acidosis (pH—7.30). An emergent ultrasound abdomen was performed which showed ill-defined bladder wall with a large mass of heterogeneous echogenicity, thought to represent clotted blood. Her plain radiograph of pelvis and contrast enhanced computed tomography revealed the left end of the K-wire reaching very close to the medial wall of left acetabulum with right end lying in the soft tissues of the pelvis away from the major vascular structures which appeared normal. There was no perivesical collection (Figures 1 and 2). Later cystoscopy was done which revealed a large bladder clot and a K-wire which was penetrating through the right lateral wall of the bladder and other end was exiting through the left lateral wall with severe inflammatory changes around the sites of penetration (Figures 3(a) and 3(b)). The bladder neck, the trigone, and the ureteric orifices were normal. After initial stabilization patient was taken for emergency laparotomy and implant was successfully extracted after a cystotomy via lower abdominal midline incision (Figures 4(a) and 4(b)). Postoperative period was uneventful. The recovery was unremarkable and patient was discharged on postoperative day 7. She was regularly followed and was doing well.

## 3. Discussion

K-wires and pins are still commonly used effective devices in the management of fractures and dislocations. The reports of migration of such implants to distant anatomical sites such as abdomen, pelvis, and even thorax are present in the literature [3]. The intrapelvic migration of these devices may lead to fatal complications due to soft tissue and vascular injuries as perforation of rectum, colon, external iliac artery, common femoral artery, bladder, and ureter, which have been already published in the literature [4–6]. The migration of K-wires has been reported between few days to years after implants. There are various proposed theories present in literature to explain such migration as muscular activity, greater motion around the joint, bone resorption secondary to prolonged implantation, chronic osteomyelitis, gravitational forces, and nonunion. Several recommendations have been made to prevent these potential complications such as bending of the wire or the use of restraining devices as screws and plates, along with restricted joint motion postoperatively [7]. Nevertheless none of these implementations completely protect the patient against pin migration. Therefore, regular clinical and radiographic followup are needed in patients with internal fixation devices. The computer tomographic scan is used to localize the exact position of implants and its relation with the surrounding structures [8]. In order to avoid any devastating complications, it is recommended that immediate removal of these devices must be considered, once desired therapeutic outcome has been achieved or any migration is recognized.

## 4. Conclusion

Intrapelvic migration of K-wires can lead to very serious and fatal complications. Thus it is an important issue to be kept in mind of surgeons dealing with patients having such implants due to previous hip surgery.

On the basis of this clinical experience, our take-home messages are

- educating the patient regarding the importance of followup;
- consideration of radiological evaluation as part of follow-up protocol;
- use of these devices must be considered only for temporary basis;
- immediate removal must be considered once desired therapeutic outcome has been achieved or any migration is noticed.
